# MEG Source Localization via Deep Learning

**DOI:** 10.3390/s21134278

**Published:** 2021-06-22

**Authors:** Dimitrios Pantazis, Amir Adler

**Affiliations:** 1 McGovern Institute for Brain Research, Massachusetts Institute of Technology, Cambridge, MA 02139, USA; pantazis@mit.edu; 2 Electrical Engineering Department, Braude College of Engineering, Karmiel 2161002, Israel

**Keywords:** magnetoencephalography, deep learning, source localization, inverse problems

## Abstract

We present a deep learning solution to the problem of localization of magnetoencephalography (MEG) brain signals. The proposed deep model architectures are tuned to single and multiple time point MEG data, and can estimate varying numbers of dipole sources. Results from simulated MEG data on the cortical surface of a real human subject demonstrated improvements against the popular RAP-MUSIC localization algorithm in specific scenarios with varying SNR levels, inter-source correlation values, and number of sources. Importantly, the deep learning models had robust performance to forward model errors resulting from head translation and rotation and a significant reduction in computation time, to a fraction of 1 ms, paving the way to real-time MEG source localization.

## 1. Introduction

Accurate localization of functional brain activity holds promise to enable novel treatments and assistive technologies that are in critical need by our aging society. The ageing of the world population has increased the prevalence of age-related health problems, such as physical injuries, mental disorders, and stroke, leading to severe consequences for patients, families, and the health care system. Emerging technologies can improve the quality of life of patients by (i) providing effective neurorehabilitation, and (ii) enabling independence in everyday tasks. The first challenge may be addressed by designing neuromodulatory interfacing systems that can enhance specific cognitive functions or treat specific psychiatric/neurological pathologies. Such systems could be driven by real-time brain activity to selectively modulate specific neurodynamics using approaches such as transcranial magnetic stimulation [1,2] or focused ultrasound [3,4]. The second challenge may be addressed by designing effective brain-machine interfaces (BMI). Common BMI control signals rely on primary sensory- or motor-related activation. However, these signals only reflect a limited range of cognitive processes. Higher-order cognitive signals, and specifically those from prefrontal cortex that encode goal-oriented tasks, could lead to more robust and intuitive BMI [5,6].

Both neurorehabilitation and BMI approaches necessitate an effective and accurate way of measuring and localizing functional brain activity in real time. This can be achieved by electroencephalography (EEG) [7,8] and MEG [9,10,11], two non-invasive electrophysiological techniques. EEG uses an array of electrodes placed on the scalp to record voltage fluctuations, whereas MEG uses sensitive magnetic detectors called superconducting quantum interference devices (SQUIDs) [12] to measure the same primary electrical currents that generate the electric potential distributions recorded in EEG. Since EEG and MEG capture the electromagnetic fields produced by neuronal currents, they provide a fast and direct index of neuronal activity. However, existing MEG/EEG source localization methods offer limited spatial resolution, confounding the origin of signals that could be used for neurorehabilitation or BMI, or are too slow to compute in real time.

Deep learning (DL) [13] offers a promising new approach to significantly improve source localization in real time. A growing number of works successfully employ DL to achieve state-of-the-art image quality for inverse imaging problems, such as X-ray computed tomography (CT) [14,15,16], magnetic resonance imaging (MRI) [17,18,19], positron emission tomography (PET) [20,21], image super-resolution [22,23,24], photoacoustic tomography [25], synthetic aperture radar (SAR) image reconstruction [26,27] and seismic tomography [28]. In MEG and EEG, artificial neural networks have been used in the past two decades to predict the location of single dipoles [29,30,31] or two dipoles [32], but generally DL methods have received little attention with the exception of a few recent studies. Cui et al. (2019) [33] used long-short term memory networks (LSTM) to identify the location and time course of a single source; Ding et. al (2019) [34] used an LSTM network to refine dynamic statistical parametric mapping solutions; and Hecker et al. (2020) [35] used feedforward neural networks to construct distributed cortical solutions. These studies are limited by the number of dipoles, or aim to address the ill-posed nature of distributed solutions.

Here, we develop and present a novel DL solution to localize neural sources comprising *multiple* dipoles, and assess its accuracy and robustness with simulated MEG data. We use the sensor geometry of the whole-head Elekta Triux MEG system and define as source space the cortical surface extracted from a structural MRI scan of a real human subject. While we focus on MEG, the same approaches are directly extendable to EEG, enabling a portable and affordable solution to source localization.

## 2. Background on MEG Source Localization

Two noninvasive techniques, MEG and EEG, measure the electromagnetic signals emitted by the human brain and can provide a fast and direct index of neural activity suitable for real-time applications. The primary source of these electromagnetic signals is widely believed to be the electrical currents flowing through the apical dendrites of pyramidal neurons in the cerebral cortex. Clusters of thousands of synchronously activated pyramidal cortical neurons can be modeled as an equivalent current dipole (ECD). The current dipole is therefore the basic element used to represent neural activation in MEG and EEG localization methods.

In this section we briefly review the notations used to describe measurement data, forward matrix, and sources, and formulate the problem of estimating current dipoles. Consider an array of *M* MEG or EEG sensors that measures data from a finite number *Q* of equivalent current dipole (ECD) sources emitting signals ${\left\{s_{q}\left(t\right)\right\}}_{q=1}^{Q}$ at locations ${\left\{p_{q}\right\}}_{q=1}^{Q}$. Under these assumptions, the M×1 vector of the received signals by the array is given by:

(1) $$y\left(t\right)=\sum_{q=1}^{Q}l\left(p_{q}\right)s_{q}\left(t\right)+n\left(t\right),$$

where $l(p_{q})$ is the topography of the dipole at location $p_{q}$ and n(t) is the additive noise. The topography $l(p_{q})$, is given by:

(2) $$l\left(p_{q}\right)=L\left(p_{q}\right)q,$$

where $L(p_{q})$ is the M×3 forward matrix at location $p_{q}$ and q is the 3×1 vector of the orientation of the ECD source. Depending on the problem, the orientation q may be known, referred to as *fixed-oriented* dipole, or it may be unknown, referred to as *freely-oriented* dipole.

Assuming that the array is sampled *N* times at $t_{1},\ldots ,t_{N}$, the matrix Y of the sampled signals can be expressed as:

(3) Y=A(P)S+N,

where Y is the M×N matrix of the received signals:

(4) $$Y=[y\left(t_{1}\right),\ldots ,y\left(t_{N}\right)],$$

A(P) is the M×Q mixing matrix of the topography vectors at the *Q* locations $P=[p_{1},\ldots ,p_{Q}]$:

(5) $$A\left(P\right)=[l\left(p_{1}\right),\ldots ,l\left(p_{Q}\right)],$$

S is the Q×N matrix of the sources:

(6) $$S=[s\left(t_{1}\right),\ldots ,s\left(t_{N}\right)],$$

with $s\left(t\right)={\left[s_{1}\left(t\right),...,s_{Q}\left(t\right)\right]}^{T}$, and N is the M×N matrix of noise:

(7) $$N=[n\left(t_{1}\right),\ldots ,n\left(t_{N}\right)].$$

**Figure 1:**
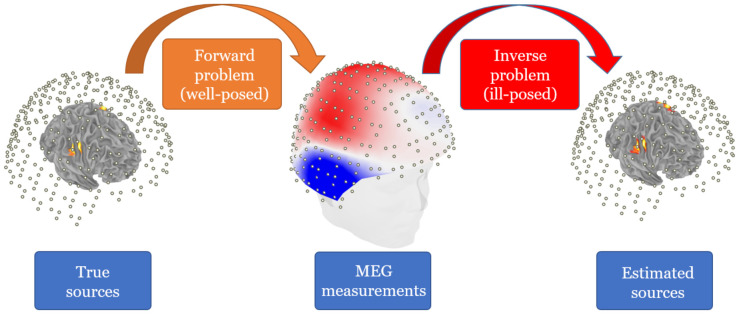
MEG forward and inverse problems. In the forward problem, a well-posed model maps the true sources activation to the MEG measurement vector. In the inverse (and ill-posed) problem, an inverse operator maps the measurement vector to the estimated sources activation.

Mathematically, the localization problem can be cast as an optimization problem of computing the location and moment parameters of the set of dipoles whose field best matches the MEG/EEG measurements in a least-squares (LS) sense [36]. In this paper we focus on solutions that solve for a small parsimonious set of dipoles and avoid the ill-posedness associated with imaging methods that yield distributed solutions, such as minimum-norm [9] (Figure 1). Solutions that estimate a small set of dipoles include the *dipole fitting* and *scanning* methods. Dipole fitting methods solve the optimization problem directly using techniques that include gradient descent, Nedler–Meade simplex algorithm, multistart, genetic algorithm, and simulated annealing [37,38,39,40]. However, these techniques remain unpopular because they converge to a suboptimal local optimum or are too computationally expensive.

An alternative approach is scanning methods, which use adaptive spatial filters to search for optimal dipole positions throughout a discrete grid representing the source space [11]. Source locations are then determined as those for which a metric (localizer) exceeds a given threshold. While these approaches do not lead to true least squares solutions, they can be used to initialize a local least squares search. The most common scanning methods are beamformers [41,42] and MUSIC [36], both widely used for bioelectromagnetic source localization, but they assume uncorrelated sources. When correlations are significant, they result in partial or complete cancellation of correlated (also referred to as synchronous) sources) and distort the estimated time courses. Several multi-source extensions have been proposed for synchronous sources [43,44,45,46,47,48,49,50,51]; however, they require some a-priori information on the location of the synchronous sources, are limited to the localization of pairs of synchronous sources, or are limited in their performance.

One important division of the scanning methods is whether they are *non-recursive* or *recursive*. The original Beamformer [41,42] and MUSIC [36] methods are non-recursive and require the identification of the largest local maxima in the localizer function to find multiple dipoles. Some multi-source variants are also non-recursive (e.g., [44,45,46,47]), and as a result they use brute-force optimization, assume that the approximate locations of the neuronal sources have been identified a priori, or still require the identification of the largest local maxima in the localizer function. To overcome these limitations, non-recursive methods have recursive counterparts, such as RAP MUSIC [52], TRAP MUSIC [53], Recursive Double-Scanning MUSIC [54], and RAP Beamformer [7]. The idea behind the recursive execution is that one finds the source locations iteratively at each step, projecting out the topographies of the previously found dipoles before forming the localizer for the current step [7,52]. In this way, one replaces the task of finding several local maxima with the easier task of finding the global maximum of the localizer at each iteration step. While recursive methods generally perform better than their non-recursive counterparts, they still suffer from several limitations, including limited performance, the need for high signal-to-noise ratio (SNR), non-linear optimization of source orientation angles and source amplitudes, or inaccurate estimation as correlation values increase. The are also computationally expensive due to the recursive estimation of sources.

## 3. Background on Deep Learning

Inverse problems in signal and image processing were traditionally solved using analytical methods; however, recent DL [13] solutions, as exemplified in [17,55], provide state-of-the-art results for numerous problems including X-ray computed tomography, magnetic resonance image reconstruction, natural image restoration (denoising, super-resolution, debluring), synthetic aperture radar image reconstruction and hyper-spectral unmixing, among others. In the following, we review DL principles, which form the basis for the DL-based solutions to MEG source localization presented in the next section, including concepts such as network layers and activation functions, empirical risk minimization, gradient-based learning, and regularization.

DL is a powerful class of data-driven machine learning algorithms for supervised, unsupervised, reinforcement and generative tasks. DL algorithms are built using Deep Neural Networks (DNNs), which are formed by a hierarchical composition of non-linear functions (layers). The main reason for the success of DL is the ability to train very high capacity (i.e., hypothesis space) networks using very large datasets, often leading to robust *representation learning* [56] and good *generalization* capabilities in numerous problem domains. Generalization is defined as the ability of an algorithm to perform well on unseen examples. In statistical learning terms, an algorithm A:X→Y is learned using a training dataset $S=\{\left(x_{1},y_{1}\right),\ldots ,\left(x_{N},y_{N}\right)\}$ of size *N*, where $x_{i}\in X$ is a data sample and $y_{i}\in Y$ is the corresponding label (for example, source location coordinates). Let P(X,Y) be the true distribution of the data, then the expected risk is defined by:

(8) $$R\left(A\right)=E_{x,y∼P(X,Y)}\left[L\left(A\left(x\right),y\right)\right],$$

where L is a loss function that measures the misfit between the algorithm output and the data label. The goal of DL is to find an algorithm A within a given capacity (i.e., function space) that minimizes the expected risk; however, the expected risk cannot be computed since the true distribution is unavailable. Therefore, the empirical risk is minimized instead:

(9) $$R_{E}\left(A\right)=\frac{1}{N}\sum_{i=1}^{N}L\left(A\left(x_{i}\right),y_{i}\right),$$

which approximates the statistical expectation with an empirical mean computed using the training dataset. The *generalization gap* is defined as the difference between the expected risk to the empirical risk: $R\left(A\right)-R_{E}\left(A\right)$. By using large training datasets and high capacity algorithms, DL has been shown to achieve a low generalization gap, where an approximation of the expected risk is computed using the learned algorithm and a held-out testing dataset $T=\{\left(x_{1},y_{1}\right),\ldots ,\left(x_{M},y_{M}\right)\}$ of size *M*, such that S∩T=∅.

In the following subsections we describe the main building blocks of DNNs, including multi-layer perceptron and convolutional neural networks.

### 3.1. Multi-Layer Perceptron (MLP)

The elementary building block of the MLP is the *Perceptron*, which computes a non-linear scalar function, termed *activation*, of an input $x\in R^{n}$, as follows

(10) $$y=f(w^{T}x+b),$$

where w is a vector of weights and *b* is a scalar bias. A common activation function is the Sigmoid [13], defined as

$$f\left(z\right)=\frac{1}{1+e^{-z}},$$

and in this case the perceptron output is computed as follows

$$y=\frac{1}{1+e^{-(w^{T}x+b)}}.$$

A single layer of perceptrons is composed of multiple perceptrons, all connected to the same input vector x, with a unique weight vector and bias, per perceptron. A single layer of perceptrons can be formulated in matrix form, as follows:

(11) y=f(Wx+b),

where each row of the matrix W corresponds to the weights of one perceptron, and each element of the vector b corresponds to the bias of one perceptron. The MLP is composed of multiple layers of perceptrons, such that the output of each layer becomes the input to the next layer. Such hierarchical composition of *k* non-linear functions is formulated as follows:

(12) $$F\left(x;\Theta \right)=f_{k}\left(f_{k-1}\left(\cdots f_{2}\left(f_{1}\left(x;\theta_{1}\right);\theta_{2}\right);\theta_{k-1}\right);\theta_{k}\right),$$

where $\theta_{i}=\left[W_{i},b_{i}\right]$ are the parameters (i.e., weights and biases) of the *i*-th layer and $\Theta =[\theta_{1},\theta_{2},\cdots ,\theta_{k}]$ is the set of all network parameters.

In our source localization solution solution for a single MEG snapshot, detailed in Section 4.1, we utilize a MLP architecture that accepts the MEG measurement vector as an input and the the final layer provides the sources locations.

In the supervised learning framework, the parameters Θ are learned by minimizing the empirical risk, computed over the training dataset S. The empirical risk can be regularized in order to improve DNN generalization, by mitigating over-fitting of the learned parameters to the training data. The empirical risk is defined by

(13) $$J\left(\Theta \right)=\frac{1}{N}\sum_{i=1}^{N}L\left(F\left(x_{i};\Theta \right),y_{i}\right),$$

and the optimal set of parameters $\Theta^{*}$ are obtained by solving

(14) $$\Theta^{*}=\arg\min_{\Theta }J\left(\Theta \right).$$

The minimization of the empirical risk is typically performed by iterative gradient-based algorithms, such as the stochastic gradient descent (SGD)

(15) $${\hat{\Theta }}_{t+1}={\hat{\Theta }}_{t}-\lambda \nabla {}_{\Theta }J\left(\Theta \right),$$

where ${\hat{\Theta }}_{t}$ is the estimate of $\Theta^{*}$ at the t-*th* iteration, λ>0 is the learning rate, and the approximated gradient $\nabla {}_{\Theta }J\left(\Theta \right)$ is computed by the *back-propagation* algorithm using a small random subset of examples from the training set S.

### 3.2. Convolutional Neural Networks

Convolutional Neural Networks (CNNs) were originally developed for processing input images, using the weight sharing principle of a convolutional kernel that is convolved with input data. The main motivation is to reduce significantly the number of required learnable parameters, as compared to processing a full image by perceptrons, namely, allocating one weight per pixel for each perceptron. A CNN is composed of one or more convolutional layers, where each layer is composed of one of more learnable kernels. For a 2D input I(i,j), a convolutional layer performs the convolution (some DL libraries implement the cross-correlation operation) between the input to the kernel(s)

(16) $$C\left(i,j\right)=\left(K*I\right)\left(i,j\right)=\sum_{m,n}W\left(m,n\right)I\left(i-m,j-n\right),$$

where W(m,n) is the kernel and C(i,j) is the convolution result. A bias *b* is further added to each convolution results, and an activation function f() is applied, to obtain the *feature map*F(i,j) given by

(17) F(i,j)=f(C(i,j)+b).

A convolutional layer with *K* kernels produces *K* feature maps, where kernels of 1D, 2D or 3D are commonly used. Convolutional layers are often immediately followed by sub-sampling layers, such as *MaxPooling* that decimates information by picking the maximum value within a given array of values, or *AveragePooling* that replaces a given array of values by their mean. CNN networks are typically composed by a cascade of convolutional layers, optionally followed by fully-connected (FC) layers, depending on the required task.

In our source localization solution using multiple MEG snapshots, detailed in Section 4.2, we utilize 1D convolutions for spatio-temporal feature extractors, which are followed by FC layers for computing the estimated sources locations.

## 4. Deep Learning for MEG Source Localization

In this section we present the proposed deep neural network (DNN) architectures and training data generation workflow.

### 4.1. MLP for Single-Snapshot Source Localization

**Figure 2:**
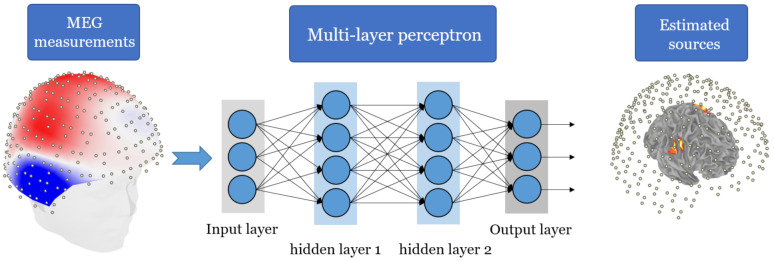
Illustration of the MLP-based MEG source localization solution. The end-to-end inversion operator performs mapping from the MEG measurement space to the source locations space.

MEG source localization is computed from sensor measurements using either a single snapshot (i.e., a single time sample) or multiple snapshots. The single snapshot case is highly challenging for popular MEG localization algorithms, such as MUSIC [36], RAP-MUSIC [52], and RAP-Beamformer [7], all of which rely on the data covariance matrix. A single snapshot estimation of the covariance matrix is often insufficient for good localization accuracy of multiple simultaneously active sources, especially in low and medium signal-to-noise ratios (SNR). Since the input in this case is a single measurement MEG vector, we implemented four layer MLP-based architectures, where the input FC layer maps the *M*-dimensional snapshot vector to a higher dimensional vector and the output layer computes the source(s) coordinates in 3D, as illustrated in Figure 2. We refer to this model as *DeepMEG-MLP*. We implemented three DeepMEG-MLP models, corresponding to Q=1,2, and 3 sources, as summarized in Table 1.

### 4.2. CNN for Multiple-Snapshot Source Localization

**Figure 3:**
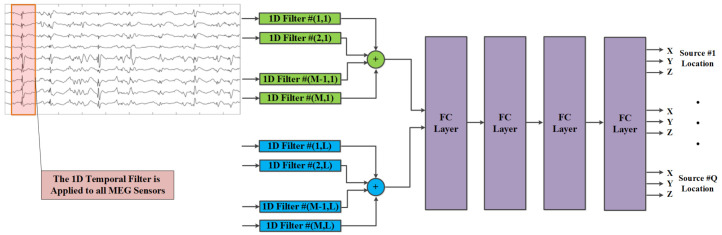
Illustration of the CNN-based MEG source localization solution. The end-to-end inversion operator performs mapping from the MEG time-series measurement space, using a bank of *L* space-time filters and four fully connected (FC) layers, to the source locations space.

For multiple consecutive MEG snapshots we implemented a CNN-based architecture with five layers, in which the first layer performs 1D convolutions on the input data and the resulting 1D feature maps are processed by three FC layers with sigmoid activation, and an output FC layer which computes the source locations. We refer to this model as *DeepMEG-CNN*, as illustrated in Figure 3. The 1D convolutional layer forms a bank of L=32 space-time filters (which can also be interpreted as beamformers [57]). Each 1D temporal filter spans T=5 time samples. A different 1D filter is applied to the time course of each of the *M* sensors with uniquely learned coefficients. We implemented three DeepMEG-CNN models, corresponding to Q=1,2, and 3 sources, as summarized in Table 1.

### 4.3. Data Generation Workflow

**Figure 4:**
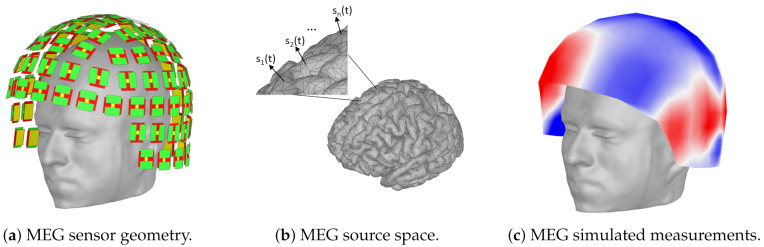
Simulation of MEG data for deep learning localization. (**a**) Simulations used the anatomy of an adult human subject and a whole-head MEG sensor array from an Elekta Triux device. (**b**) Cortical sources with time course $s_{i}\left(t\right)$ were simulated at different cortical locations. (**c**) The activated cortical sources yielded MEG measurements on the cortex that, combined with additive Gaussian noise, comprised the input to the deep learning model.

To train the deep network models and evaluate their performance on source localization, we need to know the ground truth of the underlying neural sources generating MEG data. Since this information (i.e., the true locations of the sources) is unavailable in real MEG measurements of human participants, we performed simulations with an actual MEG sensor array and a realistic brain anatomy and source configurations. Specifically, the sensor array was based on the whole-head Elekta Triux MEG system with 306-channel probe unit including 204 planar gradiometer sensors and 102 magnetometer sensors (Figure 4a). The geometry of the MEG source space was modeled with the cortical manifold extracted from a T1-weighted MRI structural scan from a real adult human subject using Freesurfer [58]. This source configuration is consistent with the arrangement of pyramidal neurons, the principal source of MEG signals, in the cerebral cortex. Sources were restricted to 15,002 grid points over the cortex (Figure 4b). The lead field matrix, which represents the forward mapping from the activated sources to the sensor array, was estimated using BrainStorm [59] based on an overlapping spheres head model [60]. This model has been shown to have accuracy similar to boundary element methods in MEG data but is orders of magnitude faster to compute [60].

Simulated MEG sensor data was generated by first activating a few sources randomly selected on the cortical manifold with activation time courses $s_{i}\left(t\right)$. The time courses were modeled with 16 time points sampled as mixtures of sinusoidal signals of equal amplitude with correlations in the range of 0 to 0.9 and frequencies in the range of 10 to 90 Hz. The corresponding sensor measurements were then obtained by multiplying each source with its respective topography vector $l(p_{i})$ (Figure 4c). Finally, Gaussian white noise was generated and added to the MEG sensors to model instrumentation noise at specific SNR levels. The SNR was defined as the ratio of the Frobenius norm of the signal-magnetic-field spatiotemporal matrix to that of the noise matrix for each trial as in [61].

## 5. Performance Evaluation

### 5.1. Deep Network Training

The DeepMEG models were implemented in TensorFlow [62] and trained by minimizing the mean-squared error (MSE) between the true (i.e., MEG data labels) and estimated sources locations, using the SGD algorithm (15) with a learning rate λ=0.001 and batch size of 32. The DeepMLP networks were trained with datasets of 1 million simulated snapshots, generated at a fixed SNR level, yet, as discussed in the following these trained models operate well in a wide range of SNR levels. The DeepCNN networks were trained using data generation on the fly and a total of 9.6 million multiple-snapshot signals per network. Data generation on the fly was utilized in order to mitigate the demanding memory requirements of offline data generation in the case of multiple-snapshots training set. The DeepCNN network models were trained with MEG sensor data at a fixed SNR-level and random inter-source correlations, thus, learning to localize sources with a wide range of inter-source correlation levels.

### 5.2. Localization Experiments

We assessed the performance of the deepMEG models using simulated data as described in the data generation workflow. To assess localization accuracy in different realistic scenarios, we conducted simulations with different SNR levels and inter-source correlation values. We also varied the number of active sources to validate that localization is accurate even for multiple concurrently active sources.

During inference, we compared the performance of the deep learning model against the popular scanning localization solution RAP-MUSIC [52]. All experiment were conducted with 1000 Monte-Carlo repetitions per each SNR and inter-source correlation value. In each scenario, we used the deepMEG and RAP-MUSIC with the corresponding number of sources, which is assumed known by both methods. Estimation of the number of sources can be conducted with the Akaike information criterion (AIC), Bayesian information criterion (BIC), or cross-validation and is beyond the scope of this work.

#### 5.2.1. Experiment 1: Performance of the DeepMEG-MLP Model with Single-Snapshot Data

**Figure 5:**
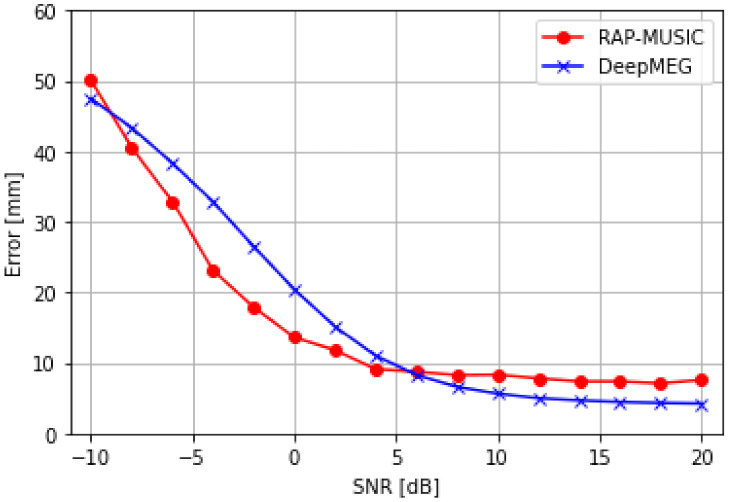
Localization accuracy of the DeepMEG-MLP model at different SNR levels for the cases of two dipole sources.

We assessed the localization accuracy of the DeepMEG-MLP model against the RAP-MUSIC method for the case of two simultaneously active dipole sources. The DeepMEG-MLP model was trained with 10 dB SNR data, but inference used different SNR levels ranging from −10 dB to 20 dB (Figure 5). The DeepMEG model outperformed the RAP-MUSIC method at high SNR values, but had worse localization results in low SNR values (<5 dB). As expected, both methods consistently improved their localization performance with increasing SNR values.

#### 5.2.2. Experiment 2: Performance of the DeepMEG-CNN Model with Multiple-Snapshot Data

**Figure 6:**
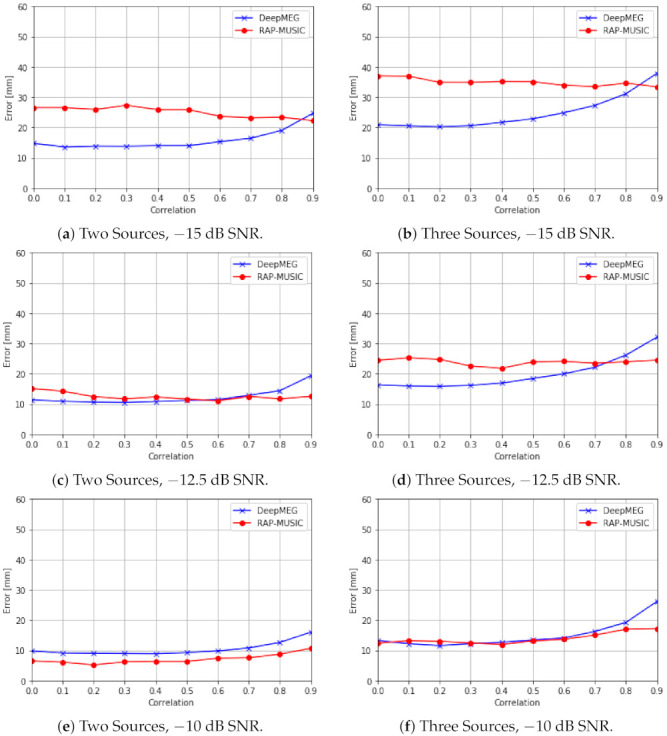
Localization accuracy of the DeepMEG-CNN model with T=16 time samples at different inter-source correlation values for the cases of two and three sources with −15 dB, −12.5 dB and −10 dB SNR levels.

We extended the above experiment for the case of multiple snapshot data with T=16 time samples and two or three sources with different inter-source correlation values. The DeepMEG-CNN model was trained with −15 dB SNR data, and inference used −15 dB, −12.5 dB, and −10 dB SNR. In the low SNR case (−15 dB), the DeepMEG-CNN consistently outperformed the RAP-MUSIC method with the exception of high (0.9) correlation values where the RAP-MUSIC had a slightly better accuracy (Figure 6a,b). As SNR increased to −12.5 dB, the Deep MEG model remained overall better or had comparable performance to RAP-MUSIC (Figure 6c,d). This advantage was lost at −10 dB SNR, where RAP-MUSIC had an advantage (Figure 6e,f).

#### 5.2.3. Experiment 3: Robustness of DeepMEG to Forward Model Errors

**Figure 7:**
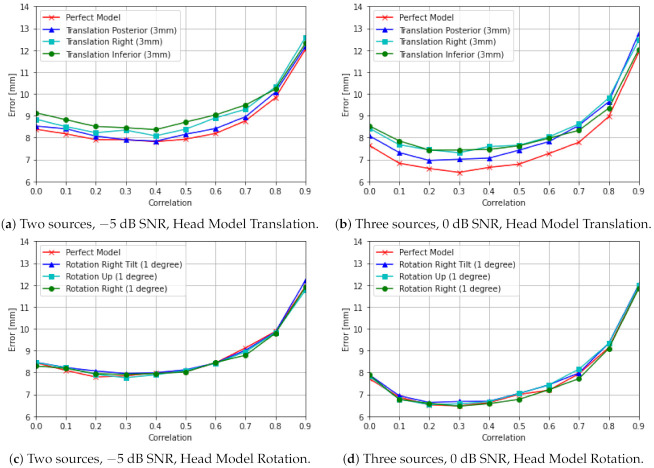
Robustness of DeepMEG-CNN localization accuracy to forward model errors due to head model translations by 3 mm (**a**,**b**), and head model rotations by 1 degree (**c**,**d**).

Here we assumed that the actual MEG forward model is different from the ideal forward model that was used for building the training set of the DeepMEG solution. The actual model was defined as follows:

(18) $$\tilde{A}\left(P\right)=A\left(P\right)+\Delta A\left(P\right),$$

where ΔA(P) denotes the *model error* matrix, which can be *arbitrary*. In this experiment we considered the model error matrix to represent inaccuracies due to imprecise estimation of the position of the head caused by translation or rotation registration errors. Figure 7 presents the DeepMEG-CNN localization accuracy with model registration errors equal to 3 mm translation and 1 degree rotation across different axes. The DeepMEG-CNN achieved robust localization accuracy in all cases: up to 1 mm degradation for head translations and up to 0.25 mm degradation for head rotations.

### 5.3. Real-Time Source Localization

An important advantage of the DL approaches is that they have significantly reduced computational time, paving the way to real-time MEG source localization solutions. We conducted a computation time comparison (compute hardware: CPU 6-Core Intel i5-9400F@3.9 Ghz, CPU RAM 64 GB, GPU Nvidia GeForce RTX 2080), detailed in Table 2, for each of the proposed DL architectures and the RAP-MUSIC algorithm. The comparison reveals 4 orders of magnitude faster computation of DeepMEG models as compared to the RAP-MUSIC, which requires O(Q)M×M matrix inversions and $O(Q\times N_{dipoles})$ matrix-vector multiplications, with each multiplication having complexity of $O(M^{2})$. Here, the total number of dipoles $N_{dipoles}=$ 15,002 and the number of sensors M=306.

## 6. Conclusions

Fast and accurate solutions to MEG source localization are crucial for real-time brain imaging, and hold the potential to enable novel applications in neurorehabilitation and BMI. Current methods for multiple dipole fitting and scanning do not achieve precise source localization because it is typically computationally intractable to find the global maximum in the case of multiple dipoles. These methods also limit the number of dipoles and temporal rate of source localization due to high computational demands. In this article, we reviewed existing MEG source localization solutions and fundamental DL tools. Motivated by the recent success of DL in a growing number of inverse imaging problems, we proposed two DL architectures for the solution of the MEG inverse problem, the DeepMEG-MLP for single time point localization, and the DeepMEG-CNN for multiple time point localization.

We compared the performance of DeepMEG against the popular RAP-MUSIC localization algorithm and showed improvements in localization accuracy in a range of scenarios with variable SNR levels, inter-source correlation values, and number of sources. Importantly, the DeepMEG inference was estimable in less than a millisecond and thus was orders of magnitude faster than RAP-MUSIC. Fast computation was possible due to the high optimization of modern DL tools, and even allows the rapid estimation of dipoles at near the 1 kHz sampling rate speed of existing MEG devices. This could facilitate the search for optimal indices of brain activity in neurofeedback and BCI tasks.

A key property of DeepMEG was its robustness to forward model errors. The localization performance of the model remained relatively stable even when introducing model errors caused by imprecise estimation of the position of the head due to translations or rotations.This is critical for real-time applications where the forward matrix is not precisely known, or movement of the subject introduces time-varying inaccuracies.

To apply the DeepMEG models in real MEG experiments, the networks must first be trained separately for each subject using the forward matrix derived from individual anatomy. Training should take place offline using simulated data, and then the trained model can be applied for inference on real-data. The amount of simulated data for training can be easily increased if necessary. To improve the validity of the predictions, the distributional assumptions of the simulated data should follow those of the experimental data as closely as possible, including the number of sources, and realistic noise distribution and shape of source time courses. Future work is needed to determine how to best specify and evaluate the impact of different distributional assumptions in the quality of the DL model predictions in different experimental settings. Once a model achieves the desirable performance, extending to new subjects could be achieved with transfer learning to fine tune the parameters with reduced computational cost.

While the DeepMEG-MLP and DeepMEG-CNN architectures yielded promising localization results, more research is needed to explore different architectures, regularizations, loss functions, and other DL parameters that may further improve MEG source localization. It is also critical to assess whether DL models remain robust to model errors under more cases of realistic perturbations, beyond the imprecise head position estimation assessed here. This includes inaccurate modeling of the source space (e.g., cortical segmentation errors); imprecise localization of sensors within the sensor array; and inaccurate estimation of the forward model (e.g., unknown tissue conductivities, analytical approximations or numerical errors) [63,64].

As both theory and simulations suggest, when the true number of sources is known, RAP-MUSIC and TRAP-MUSIC (a generalization of RAP-MUSIC) localize sources equally well [53]. Thus our localization comparisons should also extend to TRAP-MUSIC. Of course, this holds under the critical assumption that another method has assessed the true number of sources, as required by the DeepMEG-MLP and DeepMEG-CNN models. This can be done with AIC, BIC, cross-validation, or other criteria [65]. However, as extension of our work, different DL architectures could be developed to estimate both the number of sources and their location simultaneously, obviating the need to separately estimate the true number of sources.

## Figures and Tables

**Table 1:** Evaluated DeepMEG Architectures. Fully-connected layers are denoted by FC(*N*,’activation’), where *N* is the number of perdeptrons and the activation function is Sigmoid for all layers excluding output layers, which compute sources locations and do not employ an activation function. Convolutional layers are denoted by Conv1D(L,T), where *L* is the number of 1D kernels, and *T* is the length of each 1D kernel.

Input	Network	1st Layer	2nd Layer	3rd Layer	4th Layer	5th Layer	Parameters
Single MEG	MLP-1	FC (3000,’sigmoid’)	FC (2500,’sigmoid’)	FC (1200,’sigmoid’)	FC (3,’none’)	-	11,428,303
Snapshot	MLP-2	FC (3000,’sigmoid’)	FC (2500,’sigmoid’)	FC (1200,’sigmoid’)	FC (6,’none’)	-	11,431,906
	MLP-3	FC (3000,’sigmoid’)	FC (2500,’sigmoid’)	FC (1200,’sigmoid’)	FC (9,’none’)	-	11,435,509
MEG	CNN-1	Conv1D (L=32, T=5)	FC (3000,’sigmoid’)	FC (2500,’sigmoid’)	FC (1200,’sigmoid’)	FC (3,’none’)	11,711,295
Time-Series	CNN-2	Conv1D (L=32, T=5)	FC (3000,’sigmoid’)	FC (2500,’sigmoid’)	FC (1200,’sigmoid’)	FC (6,’none’)	11,714,898
	CNN-3	Conv1D (L=32, T=5)	FC (3000,’sigmoid’)	FC (2500,’sigmoid’)	FC (1200,’sigmoid’)	FC (9,’none’)	11,718,501

**Table 2:** Computation Time Comparison.

Sources	Time Samples	Algorithm	Time [ms]
1	1	RAP-MUSIC	135.47
1	1	DeepMEG MLP-1	**0.19**
2	1	RAP-MUSIC	452.17
2	1	DeepMEG MLP-2	**0.19**
3	1	RAP-MUSIC	736.76
3	1	DeepMEG MLP-3	**0.19**
1	16	RAP-MUSIC	136.59
1	16	DeepMEG CNN-1	**0.25**
2	16	RAP-MUSIC	478.23
2	16	DeepMEG CNN-2	**0.27**
3	16	RAP-MUSIC	741.51
3	16	DeepMEG CNN-3	**0.27**

## Data Availability

Not applicable.
